# MRI Voxel Morphometry Shows Brain Volume Changes in Breast Cancer Survivors: Implications for Treatment

**DOI:** 10.3390/pathophysiology32010011

**Published:** 2025-03-12

**Authors:** Alexandra Nikolaeva, Maria Pospelova, Varvara Krasnikova, Albina Makhanova, Samvel Tonyan, Aleksandr Efimtsev, Anatoliy Levchuk, Gennadiy Trufanov, Mark Voynov, Matvey Sklyarenko, Konstantin Samochernykh, Tatyana Alekseeva, Stephanie E. Combs, Maxim Shevtsov

**Affiliations:** 1 Personalized Medicine Centre, Almazov National Medical Research Centre, Akkuratova Str. 2, 197341 Saint Petersburg, Russia; shura.nicolaeva@yandex.ru (A.N.); pospelovaml@mail.ru (M.P.); krasnikova_vv@almazovcentre.ru (V.K.); a.mahanova.a@mail.ru (A.M.); samvelium@gmail.com (S.T.); atralf@mail.ru (A.E.); feuerlag999@yandex.ru (A.L.); trufanovge@mail.ru (G.T.); m.vojnov@mail.ru (M.V.); matwei.sklyarenko@yandex.ru (M.S.); neurobaby12@gmail.com (K.S.); t.alekseeva@mail.ru (T.A.); 2 Department of Radiation Oncology, Technishe Universität München (TUM), Klinikum rechts der Isar, Ismaninger Str. 22, 81675 Munich, Germany; stephanie.combs@tum.de

**Keywords:** breast cancer, breast cancer survivors, «chemobrain», brain morphometry, magnetic resonance imaging, voxel morphometry

## Abstract

Chemotherapy-related cognitive impairment termed «chemobrain» is a prevalent complication in breast cancer survivors that requires early detection for the development of novel therapeutic approaches. Magnetic resonance voxel morphometry (MR morphometry), due to its high sensitivity, might be employed for the evaluation of the early changes in the volumes of brain structures in order to explore the «chemobrain» condition. Methods: The open, prospective, single-center study enrolled 86 breast cancer survivors (43.3 ± 4.4 years) and age-matched 28 healthy female volunteers (44.0 ± 5.68). Conventional MR sequences (T1- and T2-weighted, TIRM, DWI, MPRAGE) were obtained in three mutually perpendicular planes to exclude an organ pathology of the brain. Additionally, the MPRAGE sequence was performed for subsequent MR morphometry of the volume of brain structures using the open VolBrain program. The evaluation was performed at two follow-up visits 6 months and 3 years after the completion of BC treatment. Results: According to the MR morphometry, breast cancer survivors presented with significantly decreased volumes of brain structures (including total brain volume, cerebellum volume, subcortical gray matter, etc.) as compared to healthy volunteers. Evaluation over the follow-up period of 3 years did not show the restoration of brain volume structures. Conclusions: The data obtained employing MR morphometry revealed significant reductions (that were not detected on the conventional MR sequences) in both gray and white matter in breast cancer survivors following chemotherapy. This comprehensive analysis indicated the utility of MR morphometry in detecting subtle yet statistically significant neuroanatomical changes associated with cognitive and motor impairments in patients, which can in turn provide valuable insights into the extent of structural brain alterations, helping to identify specific regions that are most affected by treatment.

## 1. Introduction

Breast cancer (BC), according to the International Agency for Research on Cancer, ranks 2nd in terms of prevalence among all oncological diseases, and ranks 1st in the world among oncological diseases in women [1]. Due to modern diagnostic approaches and active breast cancer awareness campaigns, the disease is increasingly detected in its early stages. Thus, in 2020, 72% of cases were diagnosed at stages I and II while in 2011, only 65% were detected. The incidence of advanced stages (III and IV) has decreased, with stage III cases dropping from 24.8% in 2011 to 19.6% in 2020, and stage IV cases dropping from 9.1% to 8.1%. While breast cancer is more common in older women (average age 61), there has been a rise in diagnoses among younger women, particularly in the 30–34 age range. Improved awareness, screening, and treatment have also significantly increased survival rates, with the five-year survival for breast cancer rising from 75% in the 1970s to 91% in the 2008–2014 period. The efficacy of BC treatment (surgical tumor debulking combined with chemotherapy, hormone therapy, and radiation) is determined by a multidisciplinary team and is tailored to each patient based on patient-related factors including age, hormonal status, disease stage, and tumor characteristics [2]. However, in recent studies evaluating the quality of life (QoL) of BC survivors, there is an obvious prevalence of adverse neurological effects associated with chemotherapy, including vascular complications, seizures, mood disorders, impaired cognition, and peripheral mono- and polyneuropathies. Epidemiological studies have revealed chemotherapy-induced cognitive impairment in various types of cancer patients with peripheral tumors, including breast cancer, colorectal cancer, and lymphoma, as well as brain tumors such as glioma, glioblastoma, and primary lymphoma of the central nervous system [3]. Among the various neurological side effects, some cancer survivors may experience cognitive impairment and depression, although they are usually subtle. A number of factors can either protect against cognitive impairment or put people at increased risk of it. These factors include fatigue, depression or anxiety, concomitant medication, indirect and direct effects of chemotherapy (anemia or menopause caused by chemotherapy), and patient-specific factors (e.g., age, education level, etc.) [4]. Prolonged exposure to chemotherapeutic agents leads to metabolic and structural changes in the brain and is described by the generally accepted term of «chemobrain». From the very first sessions of chemotherapy, patients note a decrease in concentration and speed of thinking, a vagueness of thoughts, and subsequently an inability to multitask [5]. Difficulties with the objective definition and measurement of «chemobrain» lead to huge differences in the estimate of the percentage of cancer survivors with «chemobrain», which ranges from 17 to 75%. Magnetic resonance imaging (MRI) is a potential method for diagnosing CNS lesions in women in the long-term following radical treatment of breast cancer. MRI is an important tool in neuroimaging, allowing for the detailed anatomical study of the structure and functional state of the brain. There are various MRI techniques, each of which has its own characteristics, advantages, and limitations. Standard MRI using T1 and T2 sequences allows for the detailed visualization of the brain structure due to its high resolution; however, it is subject to artifacts and provides only anatomical information [6]. More advanced techniques such as functional MRI (fMRI), diffusion-weighted MRI (DV-MRI), and perfusion MRI help to assess the functional activity of the brain, its microstructure, and blood supply, but are also subject to artifacts [7]. Magnetic resonance spectroscopy (MR spectroscopy), in turn, provides the possibility to assess the concentration of various metabolites in the brain, but it is quite difficult to interpret [8]. Both structural and functional MRI techniques have found their application in the study of the «chemobrain», allowing us to study all aspects of this condition [9]. In previously reported studies, diffusion tensor magnetic resonance imaging (DT-MRI) revealed microstructural changes in the tracts of the white matter of the brain in patients with postmastectomy syndrome [10]. Also, when using the fMRI method, valuable data were obtained on the features of the brain connectome of patients with vestibulo-atactic syndrome who underwent breast cancer treatment: functional connectivity in areas involved in the regulation of the coordination of movements and balance was significantly changed [11]. In addition to these techniques, there is another special MRI technique: magnetic resonance morphometry (i.e., MR morphometry, voxel morphometry). The MR morphometry method is a modern neuroimaging technique used to assess the volume of gray matter and the cerebral cortex [12]. In the process of image analysis, the brain is segmented into gray matter, white matter and cerebrospinal fluid, followed by smoothing and the possibility of reconstructing slices into three-dimensional images [13]. MR morphometry is a highly accurate method that has found wide applications in the diagnosis of Alzheimer's disease [14,15], epilepsy [16], schizophrenia [17], depression [18], and a number of other conditions that do not directly involve the central nervous system in the pathological process, such as inflammatory bowel disease [19]. Nevertheless, MRI morphometry is not yet commonly used in routine clinical practice and is primarily a research tool. In addition, MR morphometry was used to describe a decrease in gray matter volume in patients with chronic neuropathic pain who had breast cancer [20]. De Ruiter et al., in a multicenter study, demonstrated that in women after they have undergone breast cancer chemotherapy, there is a decrease in the volume of gray matter of the frontal lobe [21]. In another study by this author, a reduction in gray matter volume in the posterior parietal areas was demonstrated as a late effect of high-dose adjuvant chemotherapy [22]. We can hypothesize that similar results may be observed in these brain regions in the current study. Changes in the volumes of various brain regions are primarily associated with breast cancer treatment, as similar alterations were not observed in patients who did not undergo chemotherapy [23]. However, changes in the brain may also be associated with other breast cancer treatment approaches [24]. Thus, MR morphometry may prove to be a valuable method in assessing the characteristics of central nervous system damage in women with postmastectomy syndrome. We hypothesize that breast cancer survivors who have undergone chemotherapy experience significant and persistent reductions in brain volume, particularly in gray and white matter structures, which are not detectable with conventional MRI sequences. We further hypothesize that these volumetric changes correlate with cognitive and motor impairments observed in patients and that MR morphometry can serve as a sensitive tool for detecting such neuroanatomical alterations.

The aim of the present study was to assess the condition of the brain in patients who underwent breast cancer treatment at two time points, with the possibility of evaluating its recovery over time using MR morphometry. An open, single-center, prospective study was conducted to evaluate the volume parameters of the brain in patients in the late postoperative period after breast cancer treatment and the end of a course of chemotherapy treatment. Herein, we demonstrate that patients who underwent breast cancer treatment experienced significant changes in both gray and white matter brain volumes, as identified using MRI morphometry. These changes persisted at two time points, showing a reduction in the volumes of various brain structures, such as the cortex, subcortical gray matter, and the cerebellum. Obtained data also revealed that these changes may be associated with cognitive and motor impairments observed in patients following BC chemotherapy.

## 2. Materials and Methods

### 2.1. Patients and Healthy Volunteers

The study included 86 patients diagnosed with breast cancer who completed a course of chemotherapy treatment. The study was conducted in accordance with the principles of the Helsinki Declaration with the consent of the Ethics Committee of the Federal State Budgetary Institution “V.A. Almazov National Medical Research Center” of the Ministry of Health of the Russian Federation (dated 12 December 2022). All participants included in the study signed an informed voluntary consent form. Each of the participants passed two follow-up points in the study: (i) first, a visit within less than 6 months after the end of the course of chemotherapy; (ii) second, a visit conducted 12 months after the completion of the chemotherapeutic treatment. All participants completed both follow-up points, and no participants dropped out of the study. The average age of the patients constituted 43.27 ± 4.38 years (from 31 to 50 years) at the time of the first point of the study. All participants presented with stage II or III breast cancer who had completed a course of chemotherapy.

Inclusion criteria were the following: women aged 25 to 50 years, who have undergone radical treatment for breast cancer; clinical and neurological manifestations related to the treatment of the underlying disease; absence of complicating somatic pathology; absence of complaints regarding memory impairment, attention, or coordination issues before the onset of oncological treatment; signed informed consent. All study participants were in a state of social well-being at the time of the study. Criteria for exclusion included acute cerebrovascular accidents, myocardial infarctions, a history of traumatic brain injuries, cancers in the progressive stage, decompensated chronic diseases, mental disorders, contraindications for performance of MRI study, neurological or mental conditions in the anamnesis.

The healthy volunteer group consisted of women of similar ages, with a mean age of 44 ± 5.68 years (ranging from 28 to 46 years). Neurological examination results showed no focal or generalized neurological symptoms, and these women did not report complaints of memory or attention impairment, nor coordination issues. All participants underwent a neurological evaluation, which included collecting anamnesis data (i.e., date and type of surgery, chemotherapy and/or radiotherapy, complaints with assessment of CNS involvement, including headaches, dizziness, sleep disturbances, syncope and drop attacks, neuropsychological testing).

### 2.2. MRI Study

The study was performed on a Magnetom Vida tomograph (Siemens Healthineers, Erlangen, Germany) with a magnetic field induction force of 3 T. All participants underwent MRI examination. Considering the oncological vigilance of the patients, the study was conducted using basic pulse sequences typically employed to assess the condition of the brain in routine clinical practice. Initially, setup images were taken in three planes, obtaining low-resolution T1-weighted images using a localizer. Then, the following protocols were sequentially applied: T2_tra (2:05 s), T2_dark fluid_tra (3:56 s), DWI (3:37 s), T2_cor (2:01 s), and T1_MPRGE (5:12). These sequences are part of the standard protocols used in routine brain imaging, except for MPRAGE. In routine practice, conventional T1-WI is used. In our study, we chose not to duplicate T1-WI, instead utilizing MPRAGE results for clinical data analysis and the preparation of the study description. The main feature of the MPRAGE sequence is the high resolution; the volume of one voxel represents 1 mm^3^. The MPRAGE sequence characteristics are described in Table 1.

Image analysis was based on the post-processing of 3D MR images made with an isotropic voxel. In MR morphometry, great importance is given to postprocessing data using special mathematical algorithms and programs. MR morphometry analyses were conducted using the volBrain platform, specifically its AssemblyNet pipeline. This tool automates the segmentation and volumetric analysis of brain MRI data. The input consists of anonymized brain volumes in an NIFTI format, and the process produces detailed reports in CSV and PDF formats [25,26]. All the information that was uploaded into the segmentation program went into the template library. This library was presented with data from 30 healthy adult subjects, whose MRI data were obtained using Philips scanners: 7 cases at 1.5 Tesla and 23 cases at 3 Tesla. Its creation involved the following steps: noise removal, rough inhomogeneity correction, MNI space registration, fine inhomogeneity correction, intensity normalization, and manual labeling. The manual labeling was performed at different scales by an expert. Subsequently, the amount of data in the library was increased along the mid-sagittal plane based on the symmetric properties of the human brain. Thus, the template library has become open for all users. Currently, there is no need for manual tissue segmentation to use the template library. The algorithm for automatic tissue segmentation consists of the following steps: spatially adaptive non-local means denoising, rough inhomogeneity correction, affine registration to MNI space, fine SPM-based inhomogeneity correction, intensity normalization, Non-local Intracranial Cavity Extraction (NICE), tissue classification, non-local hemisphere segmentation (NABS), and non-local subcortical structure segmentation. Afterward, a detailed report is generated with absolute and relative volumes of various brain regions [27,28]. Accuracy of the automated segmentation was previously assessed by employing a comparative analysis of VolBrain and other similar programs with manual segmentation and it achieved high-accuracy results [26].

### 2.3. Statistical Analysis

The Statistica 12.5 program (TIBCO Software Inc., Palo Alto, CA, USA) and the GraphPad Prism 9.4.1 (GraphPad Software, La Jolla, CA, USA) programs were used for statistical analysis. Absolute and relative indicators of the number of observations were used to evaluate qualitative variables. Outliers were removed from the data before analysis using the ROUT method [29]. The analysis compared the measurement results obtained at each time point (first visit, second visit) with a group of healthy volunteers using the Mann–Whitney U-test with Dunnett corrections for multiple comparisons [30]. *p*-values less than 0.05 were considered statistically significant.

The calculation of effect sizes (Cohen's d) was performed in a free software environment R using the effect size library [31]. Spearman's r correlation coefficient calculation was used to reveal patient cognitive complaints’ linkage to regional volumetric reductions.

## 3. Results

### 3.1. Patient Characteristics

The open, single-center, prospective study enrolled 86 breast cancer patients (Table 2). The choice of tactics and the amount of treatment was determined by an oncologist for each patient individually, depending on the type of tumor, its spread, and its sensitivity to estrogen. Among the 86 patients, 57.0% underwent Madden surgery, an extensive surgical intervention with removal of the breast and axillary lymph nodes, 27.9% underwent sectoral resection, an organ-preserving operation involving the removal of only the affected fragment of the gland, and 15.1% of patients underwent subcutaneous mastectomy with simultaneous mammoplasty. The chemotherapy regimen was directly determined by the type and sensitivity of the tumor. Chemotherapy according to the FAC scheme was received by 6.9%, DOC-69.7%, AC-31.3%, CAF-6.98, and only 1.16% received treatment according to the CAP scheme.

The number of patients who underwent various combinations of treatments is presented in Table 3.

The most frequent complaints among the patients included memory decline and reduced attention span (Table 4). Thus, 69 patients (80.2%) noted memory decline, while 66 patients (76.7%) reported a reduced ability to concentrate. Difficulty finding words was experienced by 38 patients (44.2%). Out of the total number of patients, headaches among all complaints were observed in 48 patients (55.8%), dizziness was reported in 27 patients (31.4%), and syncopal episodes were recorded in 6 patients (7.0%). The dizziness experienced by the patients was not associated with a change in head position or fluctuations in blood pressure. Unsteadiness while walking was reported by 36 patients, which represents 41.9% of the total number. No significant dynamics in patient complaints were identified during the study. None of the healthy volunteers reported the above-mentioned complaints.

### 3.2. MRI Voxel Morphometry

**Figure 1:**
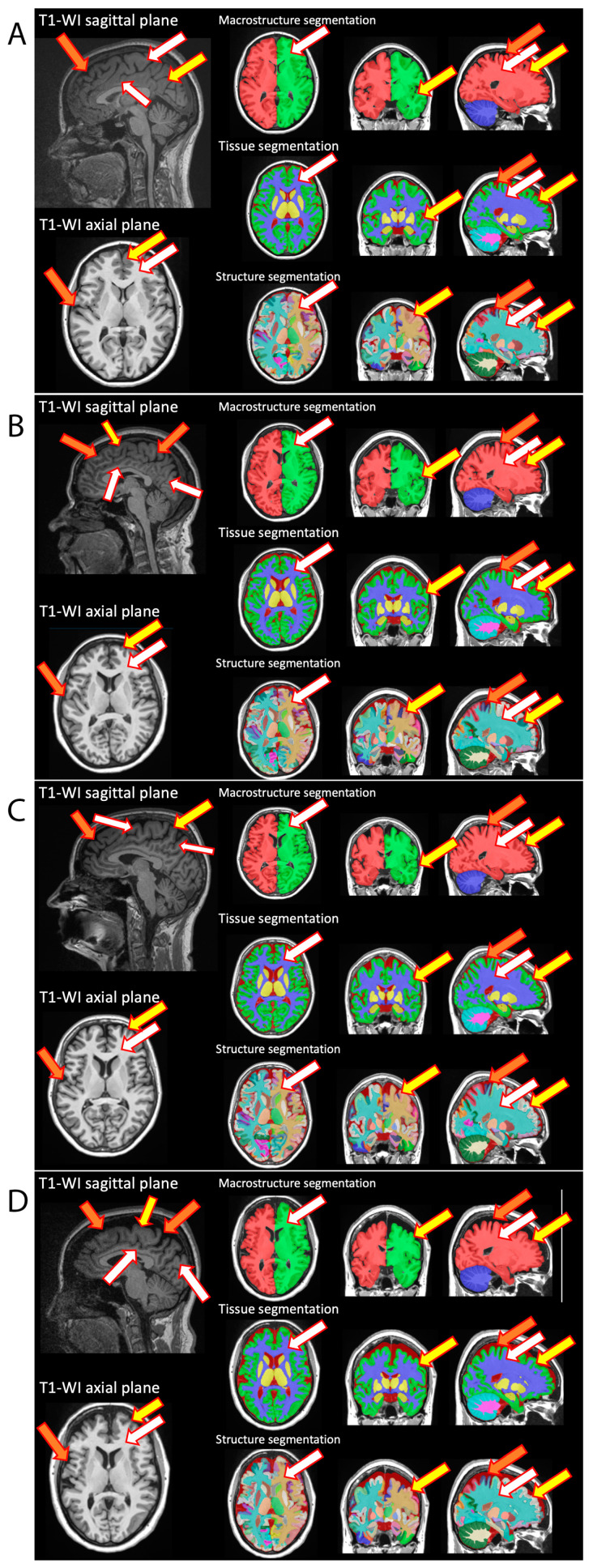
Representative MR imaging with subsequent MR voxel morphometry of healthy volunteers (**A**,**B**) and breast cancer survivors (**C**,**D**). On native MRI studies, the expansion of subarachnoid cerebrospinal fluid spaces, the deepening of furrows (orange arrow), the thinning of gray matter (yellow arrow) and a decrease in the volume of white matter (white arrow) were visualized in comparison to healthy volunteers of comparable ages. Voxel morphometry reconstructions (which included macrostructure, tissue, and structures segmentations) demonstrate the observed changes in the volumes of brain structures.

All participants, patients (*n* = 86) and healthy volunteers (*n* = 28), underwent MRI study (T1- and T2-weighted imaging, TIRM, DWI, and MPRAGE) with subsequent MRI voxel morphometry analysis of the brain structures’ volumes. Figure 1 demonstrates the representative MR images with the corresponding voxel morphometry study of healthy volunteers and breast cancer survivors following the course of chemotherapy. According to morphometry data, a difference in the volume of brain structures could be visualized including the characteristics of the gray matter of the subcortical nuclei and the white matter of the cerebellum. The red solid arrow indicates the white matter of the brain, which is indistinguishable in volume in native studies, but with a clear difference in morphometry.

Subsequent evaluation of the voxel morphometry parameters of healthy volunteers and BC survivors demonstrated the reduction in volume (cm^3^) of brain structures in the follow-up period (Table 5). At the same time, no statistically significant differences in brain volumes were identified between the first and second time points in the patients (*p* > 0.05).

**Figure 2:**
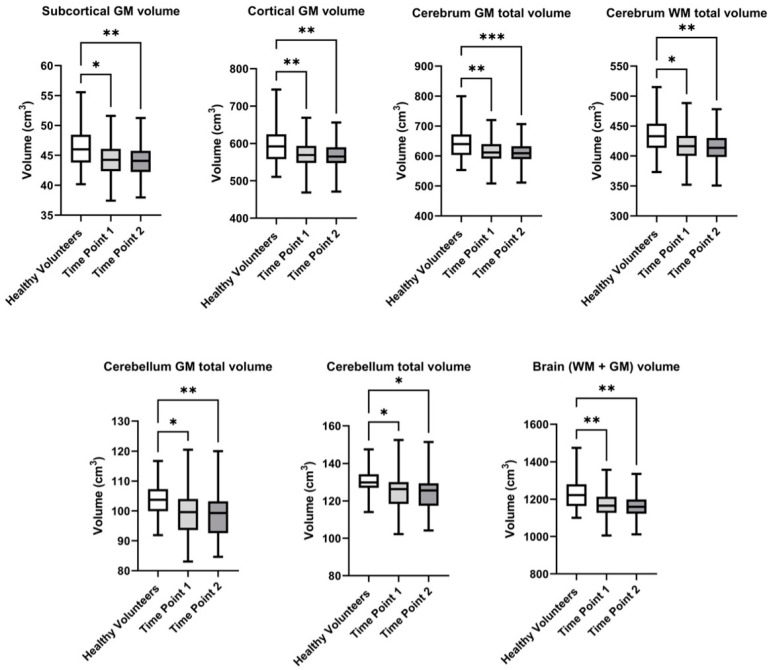
Evalution of the volume of brain structures in healthy volunteers and breast cancer survivors over the follow-up period at the first and second visits. Significant differences identified by the Mann–Whitney U-test with Dunnett corrections for multiple comparisons are shown as asterisks (* *p* < 0.05, ** *p* < 0.01, *** *p* < 0.001).

Boxplot diagrams were created to visually illustrate changes in brain structure volumes between the first and second follow-up visits, and in comparison to the healthy volunteers, making the observed differences clearer and more comprehensible (Figure 2).

**Figure 3:**
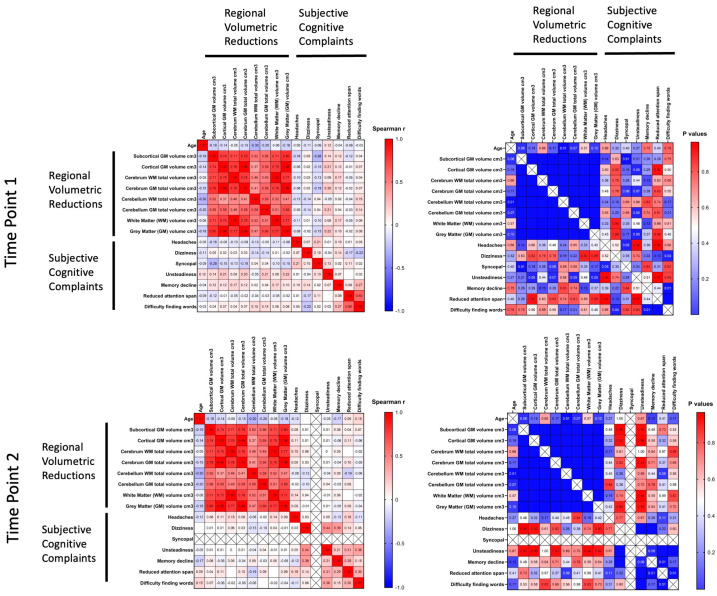
Evaluation of correlations between cognitive complaints and selected regional volumetric reductions in brain structures in breast cancer survivors over the follow-up period at the first and second visits. Spearman's r correlation coefficients with correspondent *p*-values are shown as a heat-map, from high (red) to low (blue).

Heat-maps were formed for the evaluation of the correlations between cognitive complaints and several selected regional volumetric reductions in brain structures in breast cancer survivors over the follow-up period at the first and second visits (Figure 3). The results showed a weak negative correlation with a trend toward statistical significance between a reduction in attention span and the cerebellum WM total volume.

In the control group of healthy volunteers, the brain volume (WM + GM) constituted 1221.3 ± 83.0 cm^3^, while in BC survivors, it decreased to 1165.0 ± 73.5 cm^3^ at the first visit, and further declined to 1134.2 ± 217.7 cm^3^ at the follow-up, second visit. Statistically significant differences were observed at both the first visit (*p* = 0.002) and the second visit (*p* = 0.004). A similar trend was observed when the Cerebellum GM total volume was evaluated. In healthy participants, the volume was 103.4 ± 6.4 cm^3^, whereas in patients, it was lower, measuring 98.8 ± 7.5 cm^3^ at the first visit and 96.2 ± 18.7 cm^3^ at the second visit (*p* = 0.002 and *p* = 0.004, respectively). The overall cerebellum total volume also showed statistically significant differences. Thus, it constituted 129.8 ± 8.0 cm^3^ in the control group, 124.4 ± 9.1 cm^3^ at the first visit, and decreased to 121.1 ± 23.4 cm^3^ at the follow-up (*p* = 0.003 and *p* = 0.004). A noticeable decrease was also observed in the cerebrum GM total volume, where healthy participants had a volume of 644.7 ± 52.0 cm^3^, while patients showed a reduction to 612.1 ± 38.7 cm^3^ at the first visit, and further to 595.8 ± 114.4 cm^3^ at the follow-up visit. Statistically significant differences were observed at both the first (*p* = 0.003) and second visits (*p* = 0.005). The cerebrum WM total volume also decreased from 434.7 ± 29.8 cm^3^ in the control group to 417.2 ± 33.3 cm^3^ in patients at the first visit, and to 406.4 ± 79.8 cm^3^ at the follow-up (*p* = 0.007 and *p* = 0.012). Regarding the subcortical GM, healthy participants had a volume of 46.1 ± 3.3 cm^3^, while patients had 43.8 ± 5.2 cm^3^ at the first visit, and this reduced to 43.0 ± 8.2 cm^3^ at the follow-up visit (*p* = 0.006 and *p* = 0.008). Finally, the cortical GM volume in the control group was 598.6 ± 49.4 cm^3^, whereas in patients, it was reduced to 553.3 ± 106.5 cm^3^ by the second visit (*p* = 0.004 and *p* = 0.007, respectively).

In addition to the previously described changes, statistically significant differences were identified in the following parameters. Thus, in the right basal forebrain, a volume reduction was observed as compared to healthy volunteers (*p* = 0.03). The total brain volume (including both white and gray matter) also showed statistically significant changes. In healthy volunteers, this volume constituted 1221.3 ± 83.0 cm^3^, whereas in patients, it decreased to 1165.0 ± 73.5 cm^3^ at the first visit and to 1134.2 ± 217.7 cm^3^ by the second visit (*p* = 0.004). The changes were also observed in the left calcarine cortex volume. In healthy participants, the volume was 4.5 ± 1.2 cm^3^, while in patients it decreased to 3.9 ± 0.8 cm^3^ at the first visit and remained at 3.9 ± 0.9 cm^3^ at the second visit. These changes were statistically significant, with a *p*-value of 0.011 for the first visit and 0.008 for the second. The total calcarine cortex volume also decreased from 8.8 ± 2.2 cm^3^ in healthy individuals to 8.0 ± 1.4 cm^3^ at the first visit and 7.8 ± 1.9 cm^3^ by the second visit. These differences were statistically significant, with a *p*-value of 0.024 for both visits. Finally, the left caudate volume also demonstrated significant changes. In healthy participants, the volume was 3.1 ± 0.4 cm^3^, whereas in patients, it decreased to 2.9 ± 0.5 cm^3^ at the first visit and 2.8 ± 0.6 cm^3^ by the second visit, with a *p*-value of 0.004 for both time points.

The observed voxel morphometry results clearly indicated a tendency for a reduction in both, white and gray matter volumes, in BC patients compared to the control group, with these changes being statistically significant at both the first and second follow-up visits.

## 4. Discussion

Due to the increased survival rate of cancer patients, there is an urgent need to eliminate the possible consequences that cancer treatment methods can provoke. Among these side effects, those that affect cognitive functions and other brain functions are of particular concern. The occurrence of cognitive impairment caused by chemotherapy was demonstrated in preclinical and clinical studies. In recent years, «chemobrain» has attracted attention as a serious side effect of chemotherapy. «Chemobrain» is a known condition; however, a single algorithm for diagnosing this condition has not yet been developed. Often, with moderate changes, it is difficult even for an experienced radiologist to determine the changes in the volume of any anatomical area. Earlier, Bukkieva et al. identified microstructural changes in the tracts of the white matter of the brains of breast cancer survivors using diffusion tensor imaging (DTI) [10]. The authors described a decrease in fractional anisotropy in the brain vault between the hippocampus, mastoids, and thalamus that could potentially have an influence on long- and short-term memory [10]. In a more recently reported study, Nikolaeva et al. evaluated the functional connectivity of various brain regions and the correlation with the level of biomarkers of CNS and endothelial damage in a group of patients following breast cancer treatment. The authors were able to identify two subgroups among the patients which included the patients with impaired coordination and those without. At the same time, a statistically significant decrease in functional connectivity was revealed in the areas responsible for the regulation of positional reflexes and balance, which positively correlated with an increase in the level of biomarkers of central nervous system and endothelial damage in the group of patients with imbalance [11]. In the current study, for the assessment of the volume of brain structures, the technique of MR voxel morphometry (with the use of the open VolBrain program) was employed. It allows a radiologist to estimate the absolute volume of a particular brain structure and compare it with reference values, including determining the volumes of the intracranial cavity (the sum of all indicators of gray, white matter (GM, WM) and cerebrospinal fluid (CSF)), the isolated CSF, GM, WM, and the volumes of individual structures and departments of the brain (the separation of the left and right hemispheres and cerebellum, brain stem, ventricles, and subcortical structures) [32,33]. The program is actively used in neuroimaging research, allowing for the comparison of various image processing and analysis tools [34]. The VolBrain software package makes it possible to evaluate the study of a single subject due to the presence in the program database of an extensive library of more than 600 subjects of different genders and ages [26]. In the current study, the gender and age of the breast cancer survivors (*n* = 86) was indicated in order for the VolBrain algorithms to provide population normal volumes and asymmetry boundaries for all studied brain structures. Additionally, in the present study, a group of healthy volunteers was used for reference values for MR morphometry. A statistically significant difference in the brain structures’ volumes of the studied BC survivors compared to the control group was demonstrated over the follow-up period of 3 years. Evaluated patients complained of decreased concentration, decreased memory, restlessness when walking, dizziness, and fainting (Table 3). Based on the data analysis, we observed a statistically significant decrease in total brain volume (WM + GM), with a *p*-value of 0.002 for the first visit and 0.004 for the second visit, indicating consistent volumetric reductions in both white and gray matter over time. Similarly, there was a reduction in subcortical gray matter volume (*p* = 0.008), suggesting an ongoing decrease in deeper brain structures associated with cognitive and emotional processing. The cortical gray matter volume also demonstrated significant reductions (*p* = 0.007), highlighting structural changes in the cerebral cortex, which might be linked to cognitive impairments such as memory and attention deficits. Additionally, the total white matter volume of the cerebrum showed a statistically significant decrease (*p* = 0.012), indicating plausible disruptions in neuronal connectivity and information processing. Further analysis revealed a notable decrease in cerebrum gray matter volume (*p* = 0.005), reinforcing the impact on overall gray matter integrity. In the cerebellar gray matter, the reductions were particularly prominent (*p* = 0.004), reflecting significant volumetric changes in regions associated with motor coordination and balance. In the anterior cingulate gyrus (right), the volume decreased significantly (*p* = 0.010), reflecting changes in regions involved in cognitive control. We were unable to identify a correlation between the patients’ complaints and the morphometry data. However, the brain structures whose volumes were reduced undoubtedly play an important functional role. This issue requires further investigation, as studies on Alzheimer's disease have shown a direct correlation between cognitive decline and the reduction in brain structure volumes according to MR morphometry [14,35]. In addition, the correlation between brain volume in breast cancer patients who underwent chemotherapy and cognitive functions was also found in the limbic system in the study by Chin-Hung Chen et al. [36]. Previously, Banker et al. described the significance of the precentral gyrus, the neocortex of which consists of six-word clusters, from which many cortico-spinal, corticubulbar, and cortico-rubrospinal tracts begin, as well as short associative fibers with neighboring structures [37]. Furthermore, Silva et al. indicated the importance of the precentral gyrus not only for the localization of Broca's area, as a center for planning movements in speech production, but also for the evaluation of the characteristics of perceived sound, such as pitch changes. Various departments of the precentral gyrus respond to listening and repetition, and during a period of silent delay, they hold and potentially plan a word before verbalizing it [38]. The cortex of the superior frontal gyrus is no less significant. El-Baba et al. established that the superior frontal gyrus of the dominant hemisphere is a key component in working memory as well as spatial information processing. At the same time, the superior frontal gyrus of the subdominant hemisphere participates in impulse control and its activation forms inhibitory control and motor activity [39].

The functions of the cerebellum were also described in detail in the study of Jimsheleishvili et al. that demonstrated the involvement of the cerebellum in gait coordination, the maintaining of posture and muscles’ tone. At the same time, if the cortex of the cerebellar vermis is responsible for coordinating the movements of the trunk [40], then the cortex of the hemispheres provides planning for sequential movements of the entire body [41]. Indeed, a significant lesion of all these structures will lead to vivid clinical manifestations such as ataxia, dysmetria, dizziness [42], alexia, agnosia, apraxia [43], changes in psyche and behavior, and cognition states including apathy and abulia [44]. The long-term effect of chemotherapy further aggravates these changes in the CNS. Despite significant advances in understanding the mechanisms of «chemobrain» formation, both at the clinical level and at the cellular and molecular bases, there are currently no validated or approved methods and/or tests for the diagnosis of such brain conditions. It is very heterogeneous and has many concomitant factors, such as genetics, treatment regimen, and comorbidity with other neurological conditions.

In the future, we will further expand the study subgroup depending on the type of chemotherapeutic regimens, since many authors note the connection between a specific chemotherapy drug and certain manifestations. Thus, Chen et al. noted that in the group treated with cyclophosphomide, a decrease in the volume of the temporal lobe was observed as compared to healthy volunteers [45]. Our data do not contradict the results of other studies. Inagaki et al., in a group of breast cancer survivors following chemotherapy, revealed statistically significant differences in some areas of the brain when comparing the group with and without chemotherapy. Nevertheless, after 3 years, these changes were no longer detected [46]. There are other studies describing the recovery of brain flow a few months after the end of chemotherapy. Thus, McDonald and colleagues described a partial restoration of gray matter density a year after the end of treatment for women [47]. In our study, we assessed the brain conditions of women who were 31–50 years old. We were able to trace the state of the brain at two points with a difference of 2.5–3 years from the first visit. Unlike the study of McDonald et al., we were unable to trace even a partial restoration of the structure of the brain. On the contrary, its volumes only decreased both when comparing first and second visits (as well as compared to a group of healthy volunteers), which confirms the effect of chemotherapy on the central nervous system regardless of age. The question of neuroplasticity and the ability to restore the brain is also of interest, namely, whether the brain of a young woman will recover faster compared to the brain of an older woman and what external factors and internal resources will contribute to this, or maybe whether the brains of younger people are more susceptible to the influence of chemotoxic agents than the brains of older generations.

Despite a relatively small sample of studied patients, we were able to identify statistically significant changes in the volumes of brain structures. The main limitation of the current study is that all the studied breast cancer survivors were assessed following complex treatment and a course of chemotherapy, without the MR data prior to the therapies, and due to the small sample size, it was not feasible to divide the participants into even smaller groups based on different treatment regimens. Thus, Henneghan et al. showed that cortical thinning is observed when comparing MRI data before and after a course of chemotherapy [48]. In the future, further increasing the duration of the follow-up period of observation as well as MR assessment prior to the therapies might help to detect the possible restoration of volume structures and identify factors affecting it.

## 5. Conclusions

In breast cancer survivors, changes in the central nervous system were detected following the complex treatment of breast cancer that included surgical tumor debulking with subsequent radiochemotherapy. Thus, following the complex treatment of breast cancer, patients showed a statistically significant decrease in the volume of certain brain structures as compared to the group of healthy female volunteers of the same age. When analyzing data on the state of the brain at two different time periods with an interval of 2.5–3 years, we did not find statistically significant positive dynamics in restoring brain volumes in patients. The application of the MR voxel volumetry of the brain structures provided the possibility to detect even minimal changes in the volume parameters of the brain that were not reported on the conventional MR sequences. These findings highlight the importance of incorporating MR morphometry into clinical practice for the early detection and monitoring of structural brain changes in breast cancer survivors. The ability to identify subtle neuroanatomical alterations could aid in the development of targeted rehabilitation strategies aimed at mitigating cognitive and motor deficits.

## Figures and Tables

**Table 1:** Parameters of the MPRAGE pulse sequence used in the presented study.

	T2_tra	T2_tirm	DWI	T2_cor	T1_MPRAGE
Repetition time/TR	3970.0 ms	9000.0 ms	2800.0 ms	3500.0 ms	2300 ms
Echo time/TE	95.00 ms	96.0 ms	79.00 ms	95.00 ms	2.98 ms
FoV	220 mm	220 mm	220 mm	220 mm	256 mm
Slice thickness	4.0 mm	4.0 mm	3.0 mm	4.0 mm	1.2 mm
Voxel size × (mm), y (mm)	0.4 × 0.4 × 4.0 mm	0.7 × 0.7 × 4.0 mm	1.7 × 1.7 × 3.0 mm	0.2 × 0.2 × 4.0 mm	1.0 × 1.0 × 1.1 mm
Study time	2:05	3:56	3:37	2:01	5:12

**Table 2:** Breast cancer survivors involved in the study.

	Patients (*n*)	Age (years)	Scope of Surgery			Chemotherapy					Hormone Therapy	Radiotherapy
			According to Madden	Sectoral Resection	Subcutaneous Mastectomy with Single-Stage Mammoplasty	FAC	DOC	AC	CAP	CAF	Tamoxifen	
Patients	86 (100%)	43.27 ± 4.38	49 (57.0%)	24 (27.9%)	13 (15.1%)	6 (6.9%)	60 (69.7%)	27 (31.3%)	1 (1.16%)	6 (6.98%)	51 (59.3)	57 (66.2%)
Healthy volunteers	28	44 ± 5.68	-	-	-	-	-	-	-	-	-	-

Abbreviations: FAC—fluorouracil, adriamycin, cyclophosphamide; DOC—docetaxel, paclitaxel; CAP—cyclophosphamide, adriamine; CAF—cyclophosphamide, adriamycin, ftorouracil; AC—cyclophosphamide, adriamycin.

**Table 3:** Distribution of patients by treatment combination.

Treatment Combination	Number of Patients	Percentage (%)
Chemotherapy + Hormone Therapy + Radiotherapy	41	47.9
Chemotherapy + Hormone Therapy	11	12.1
Chemotherapy + Radiotherapy	24	28.4
Hormone Therapy + Radiotherapy	5	5.8
Chemotherapy	5	5.8
Hormone Therapy	0	0.0
Radiotherapy	0	0.0

**Table 4:** The main clinical presentations of breast cancer survivors.

Clinical Presentation	Patients, *n*	Percentage (%)
Headaches	48	55.8
Dizziness	27	31.4
Syncopal	6	7
Unsteadiness while walking	36	41.9
Memory decline	69	80.2
Reduced attention span	66	76.7
Difficulty finding words	38	44.2

**Table 5:** Comparison of brain structure volumes (cm^3^) between healthy volunteers and breast cancer survivors. Data are presented as mean (M) ± standard deviation (SD). Differences identified by the Mann–Whitney U-test with Dunnett corrections for multiple comparisons (*p*-value) and standardized effect sizes (Cohen's d) are shown.

Region of Interest	Healthy Volunteers, Volume (cm^3^)	Breast Cancer Survivors		Statistical Analysis	
		First Visit (cm^3^)	Second Visit (cm^3^)	*p*-Value, Cohen's d (First Visit vs. Healthy Volunteers)	*p*-Value, Cohen's d (Second Visit vs. Healthy Volunteers)
Amygdala, left	1.1 ± 0.1	1.0 ± 0.1	1.0 ± 0.2	0.008, 0.39	0.001, 0.56
Amygdala, total	2.2 ± 0.2	2.1 ± 0.3	2.0 ± 0.4	0.085, 0.31	0.016, 0.38
Anterior cingulate gyrus, right	5.5 ± 1.3	4.9 ± 0.8	4.8 ± 1.1	0.009, 0.34	0.010, 0.34
Anterior insula, left	4.4 ± 0.4	4.1 ± 0.5	4.0 ± 0.8	0.004, 0.51	0.002, 0.53
Anterior insula, right	4.3 ± 0.4	4.0 ± 0.4	3.9 ± 0.8	0.007, 0.61	0.004, 0.64
Anterior insula, total volume	8.7 ± 0.7	8.1 ± 0.9	7.9 ± 1.6	0.003, 0.59	0.002, 0.61
Basal forebrain, right	0.3 ± 0.1	0.3 ± 0.0	0.3 ± 0.1	0.029, 0.65	0.030, 0.65
Brain (WM + GM)	1221.3 ± 83.0	1165.0 ± 73.5	1134.2 ± 217.7	0.002, 0.47	0.004, 0.74
Calcarine cortex, left	4.5 ± 1.2	3.9 ± 0.8	3.9 ± 0.9	0.011, 0.30	0.008, 0.25
Calcarine cortex, total volume	8.8 ± 2.2	8.0 ± 1.4	7.8 ± 1.9	0.024, 0.25	0.024, 0.26
Caudate, left	3.1 ± 0.4	2.9 ± 0.5	2.8 ± 0.6	0.004, 0.55	0.004, 0.73
Caudate, right	3.2 ± 0.4	2.9 ± 0.5	2.9 ± 0.7	0.003, 0.52	0.003, 0.67
Caudate, total volume	6.3 ± 0.8	5.8 ± 0.9	5.6 ± 1.3	0.003, 0.54	0.004, 0.70
Cerebellar GM	115.5 ± 8.0	110.1 ± 8.2	107.3 ± 20.8	0.002, 0.35	0.004, 0.44
Cerebellum GM, left	51.0 ± 3.4	48.9 ± 3.6	47.6 ± 9.2	0.004, 0.30	0.009, 0.39
Cerebellum GM, right	52.3 ± 3.2	49.9 ± 4.0	48.6 ± 9.5	0.001, 0.40	0.002, 0.51
Cerebellum GM, total volume	103.4 ± 6.4	98.8 ± 7.5	96.2 ± 18.7	0.002, 0.36	0.004, 0.46
Cerebellum, left	64.3 ± 4.1	61.7 ± 4.4	60.1 ± 11.6	0.004, 0.41	0.007, 0.44
Cerebellum, right	65.5 ± 4.2	62.7 ± 4.7	61.0 ± 11.8	0.003, 0.53	0.005, 0.55
Cerebellum, total volume	129.8 ± 8.0	124.4 ± 9.1	121.1 ± 23.4	0.003, 0.48	0.004, 0.51
Cerebrum GM, left	322.3 ± 26.3	305.0 ± 19.2	297.0 ± 57.1	0.002, 0.55	0.004, 0.78
Cerebrum GM, right	322.3 ± 25.8	307.0 ± 19.7	298.7 ± 57.4	0.005, 0.44	0.009, 0.65
Cerebrum GM, total volume	644.7 ± 52.0	615.9 ± 39.9	612.1 ± 38.7	0.003, 0.83	0.0007, 0.83
Cerebrum, left	540.4 ± 39.0	513.4 ± 33.4	499.9 ± 96.3	0.002, 0.51	0.003, 0.80
Cerebrum, right	539.0 ± 38.3	515.9 ± 33.8	502.1 ± 96.6	0.004, 0.39	0.008, 0.68
Cerebrum, total volume	1079.4 ± 77.1	1029.3 ± 67.1	1002.1 ± 192.9	0.003, 0,45	0.004, 0.74
Cerebrum WM, left	218.0 ± 15.2	208.3 ± 16.1	203.0 ± 39.7	0.004, 0.40	0.007, 0.74
Cerebrum WM, right	216.6 ± 14.6	208.9 ± 17.4	203.5 ± 40.1	0.013, 0.24	0.021, 0.67
Cerebrum WM, total volume	434.7 ± 29.8	418.2 ± 29.8	415.4 ± 29.1	0.029, 0.32	0.009, 0.71
Cortical GM	598.6 ± 49.4	571.9 ± 38.1	568.3 ± 37.03	0.004, 0.67	0.001, 0.77
Cuneus, left	5.3 ± 1.0	4.9 ± 0.8	4.8 ± 1.1	0.046, 0.37	0.073, 0.65
Cuneus, right	5.6 ± 1.1	5.1 ± 0.8	5.0 ± 1.2	0.053, 0.55	0.049, 0.62
Cuneus, total volume	10.9 ± 1.9	9.9 ± 1.5	9.7 ± 2.2	0.015, 0.49	0.019, 0.72
Frontal lobe, left	98.1 ± 9.3	91.7 ± 7.3	89.4 ± 17.5	0.001, 0.44	0.002, 0.64
Frontal lobe, right	97.2 ± 10.5	92.8 ± 7.3	90.5 ± 17.7	0.016, 0.32	0.021, 0.42
Frontal lobe, total volume	195.3 ± 19.3	184.5 ± 13.9	179.9 ± 35.0	0.004, 0.40	0.007, 0.53
Gray matter (GM)	760.1 ± 57.2	722.2 ± 44.0	703.1 ± 134.7	0.002, 0.49	0.004, 0.71
Gyrus rectus, left	1.9 ± 0.3	1.8 ± 0.3	1.8 ± 0.4	0.037, 0.03	0.153, 0.24
Inf. occipital gyrus, left	7.7 ± 1.6	6.8 ± 1.2	6.7 ± 1.6	0.028, 0.78	0.029, 0.93
Inf. temporal gyrus, left	13.7 ± 1.6	12.7 ± 1.5	12.3 ± 2.6	0.013, 0.47	0.004, 0.43
Inf. temporal gyrus, right	13.6 ± 2.1	12.6 ± 1.4	12.3 ± 2.5	0.021, 0.59	0.024, 0.61
Inf. temporal gyrus, total volume	27.3 ± 3.1	25.3 ± 2.5	24.5 ± 4.9	0.006, 0.62	0.003, 0.61
Insular cortex, left	15.4 ± 1.4	14.7 ± 1.7	14.3 ± 3.0	0.043, 0.27	0.033, 0.28
Intracranial cavity (IC)	1425.3 ± 104.3	1359.0 ± 95.7	1324.5 ± 256.7	0.003, 0.39	0.007, 0.69
Lateral orbital gyrus, left	2.7 ± 0.6	2.5 ± 0.5	2.4 ± 0.6	0.020, 0.34	0.003, 0.34
Lateral orbital gyrus, right	2.6 ± 0.6	2.4 ± 0.5	2.3 ± 0.6	0.030, 0.31	0.013, 021
Lateral orbital gyrus, total volume	5.4 ± 0.7	4.9 ± 0.9	4.7 ± 1.1	0.008, 0.42	0.001, 0.37
Lateral ventricle, left	9.4 ± 3.2	8.4 ± 5.2	8.5 ± 5.4	0.013, 0.25	0.019, 0.13
Lateral ventricle, total volume	17.7 ± 6.0	16.4 ± 9.2	16.6 ± 9.6	0.047, 0.16	0.060, 026
Limbic cortex, right	21.8 ± 2.6	20.4 ± 2.0	19.9 ± 4.0	0.006, 0.17	0.007, 0.39
Lobules VI-VII	2.8 ± 0.4	2.6 ± 0.3	2.6 ± 0.5	0.023, 0.37	0.026, 0.39
Medial orbital gyrus, left	4.9 ± 0.7	4.5 ± 0.6	4.5 ± 1.0	0.007, 0.16	0.021, 0.52
Middle occipital gyrus, left	6.3 ± 1.5	5.5 ± 1.1	5.4 ± 1.4	0.012, 0.52	0.010, 0.83
Middle occipital gyrus, total	12.1 ± 1.9	11.2 ± 1.5	10.9 ± 2.3	0.074, 0.44	0.044, 0.70
Occipital lobe, left	45.2 ± 5.7	41.8 ± 3.7	40.9 ± 8.1	0.003, 0.50	0.004, 0.74
Occipital lobe, total volume	90.8 ± 10.8	85.3 ± 6.8	83.1 ± 16.3	0.009, 0.47	0.013, 0.71
Opercular inf. frontal gyrus, right	3.9 ± 1.1	3.4 ± 0.7	3.3 ± 0.8	0.032, 0.63	0.016, 0.51
Opercular inf. frontal gyrus, total volume	7.3 ± 1.2	6.7 ± 1.1	6.5 ± 1.5	0.018, 0.75	0.018, 0.70
Orbital inf. frontal gyrus, right	1.6 ± 0.5	1.4 ± 0.4	1.4 ± 0.4	0.070, 0.22	0.029, 0.02
Parietal lobe, left	58.6 ± 5.5	55.8 ± 7.5	54.6 ± 12.0	0.005, 0.28	0.012, 1.02
Parietal lobe, right	59.9 ± 5.0	56.9 ± 6.7	55.5 ± 11.7	0.004, 0.29	0.007, 0.93
Parietal lobe, total volume	118.4 ± 10.2	112.7 ± 14.1	110.1 ± 23.6	0.003, 0.29	0.006, 1.00
Planum polare, left	2.2 ± 0.5	2.0 ± 0.3	2.0 ± 0.4	0.025, 0.58	0.028, 0.67
Postcentral gyrus medial segment, right	1.3 ± 0.3	1.1 ± 0.3	1.1 ± 0.4	0.018, 0.67	0.018, 0.87
Postcentral gyrus medial segment, total volume	2.4 ± 0.5	2.1 ± 0.5	2.0 ± 0.6	0.011, 0.59	0.012, 0.81
Postcentral gyrus, right	12.1 ± 1.7	11.0 ± 1.2	10.9 ± 2.2	0.002, 0.53	0.005, 0.88
Postcentral gyrus, total volume	25.0 ± 3.0	23.0 ± 2.1	22.6 ± 4.5	0.001, 0.78	0.003, 1.09
Posterior cingulate gyrus, right	5.3 ± 0.7	4.8 ± 0.8	4.8 ± 1.1	0.001, 0.03	0.003, 0.36
Posterior cingulate gyrus, total volume	10.3 ± 1.4	9.7 ± 1.4	9.5 ± 2.1	0.030, 0.06	0.035, 0.21
Posterior insula, left	2.4 ± 0.3	2.2 ± 0.3	2.2 ± 0.5	0.008, 0.52	0.013, 0.60
Posterior insula, right	2.5 ± 0.3	2.3 ± 0.3	2.3 ± 0.5	0.054, 0.34	0.041, 0.20
Posterior insula, total volume	4.9 ± 0.5	4.5 ± 0.6	4.4 ± 0.9	0.009, 0.45	0.016, 0.43
Precentral gyrus, left	14.3 ± 1.8	13.4 ± 1.2	13.1 ± 2.6	0.010, 0.31	0.020, 0.54
Precentral gyrus medial segment, right	3.1 ± 0.5	2.8 ± 0.5	2.8 ± 0.7	0.004, 0.41	0.007, 0.60
Precentral gyrus medial segment, total volume	6.1 ± 1.1	5.6 ± 0.9	5.5 ± 1.3	0.029, 0.38	0.031, 0.50
Precentral gyrus, right	14.0 ± 1.9	13.5 ± 1.3	13.3 ± 2.6	0.031, 0.03	0.044, 0.28
Precentral gyrus, total volume	28.3 ± 3.5	26.9 ± 2.3	26.4 ± 5.2	0.010, 0.16	0.025, 042
Precuneus, left	12.1 ± 1.6	11.4 ± 1.4	11.2 ± 2.3	0.031, 0.40	0.045, 0.70
Putamen, left	4.5 ± 0.4	4.3 ± 0.6	4.2 ± 0.9	0.018, 0.54	0.020, 0.63
Putamen, right	4.5 ± 0.4	4.3 ± 0.6	4.2 ± 0.9	0.020, 0.56	0.017, 0.66
Putamen, total	9.1 ± 0.8	8.6 ± 1.2	8.4 ± 1.9	0.019, 0.55	0.016, 0.65
Subcortical GM	46.1 ± 3.3	44.4 ± 3.0	43.8 ± 5.2	0.095, 0.67	0.018, 0.77
Sup. frontal gyrus, left	16.3 ± 2.1	15.0 ± 2.4	14.6 ± 3.4	0.002, 0.40	0.001, 1.02
Sup. frontal gyrus medial segment, left	6.8 ± 1.1	6.2 ± 1.0	6.1 ± 1.4	0.033, 0.55	0.026, 0.49
Sup. frontal gyrus, right	15.9 ± 2.2	14.6 ± 1.8	14.3 ± 3.0	0.009, 0.62	0.013, 0.69
Sup. frontal gyrus, total volume	32.2 ± 3.9	29.6 ± 3.8	28.9 ± 6.2	0.002, 0.54	0.003, 0.91
Sup. occipital gyrus, left	4.9 ± 0.8	4.2 ± 0.8	4.1 ± 1.0	0.001, 0.59	0.001, 1.00
Sup. occipital gyrus, total volume	10.0 ± 1.4	8.9 ± 1.3	8.7 ± 1.9	0.001, 0.61	0.001, 1.07
Sup. parietal lobule, left	12.4 ± 1.5	11.9 ± 2.5	11.6 ± 3.0	0.032, 0.07	0.030, 0.36
Sup. parietal lobule, right	12.7 ± 1.4	12.0 ± 1.9	11.7 ± 2.7	0.011, 0.07	0.005, 0.43
Sup. parietal lobule, total volume	25.1 ± 2.5	23.9 ± 4.2	23.3 ± 5.7	0.014, 0.01	0.011, 0.42
Sup. temporal gyrus, left	7.9 ± 1.0	7.1 ± 0.7	6.9 ± 1.4	0.002, 0.94	0.001, 1.00
Sup. temporal gyrus, total volume	15.3 ± 2.0	14.0 ± 1.6	13.6 ± 2.8	0.020, 0.72	0.018, 0.81
Supplementary motor cortex, left	6.0 ± 1.0	5.6 ± 0.7	5.5 ± 1.1	0.011, 0.19	0.018, 0.37
Supplementary motor cortex, total volume	11.6 ± 1.5	10.9 ± 1.4	10.7 ± 2.3	0.040, 0.24	0.049, 0.39
Supramarginal gyrus, total volume	19.3 ± 3.1	18.0 ± 2.5	17.6 ± 3.9	0.037, 0.53	0.041, 0.77
Temporal lobe, left	60.6 ± 4.9	57.5 ± 4.1	55.9 ± 10.8	0.010, 0.54	0.011, 0.63
Temporal lobe, right	60.0 ± 4.9	57.4 ± 4.5	55.7 ± 10.8	0.018, 0.32	0.012, 0.46
Temporal lobe, total volume	120.6 ± 9.5	115.0 ± 8.3	111.6 ± 21.6	0.011, 0.44	0.011, 0.56
Thalamus, left	8.1 ± 0.8	7.6 ± 0.9	7.4 ± 1.6	0.006, 0.53	0.007, 0.71
Thalamus, right	8.1 ± 0.7	7.7 ± 0.9	7.5 ± 1.6	0.020, 0.41	0.032, 0.60
Thalamus, total	16.2 ± 1.5	15.3 ± 1.9	15.0 ± 3.2	0.010, 0.47	0.017, 0.66
Triangular inf. frontal gyrus, left	4.1 ± 0.9	3.7 ± 0.7	3.6 ± 0.9	0.041, 0.73	0.028, 0.33
Ventral DC, left	5.0 ± 0.4	4.7 ± 0.3	4.6 ± 0.9	0.003, 0.90	0.004, 1.11
Ventral DC, right	4.9 ± 0.4	4.6 ± 0.4	4.4 ± 0.9	0.001, 0.91	0.001, 1.23
Ventral DC, total volume	9.8 ± 0.9	9.3 ± 0.7	9.0 ± 1.7	0.001, 0.95	0.001, 1.19
Vermis	12.1 ± 2.3	11.3 ± 1.0	11.1 ± 2.2	0.025, 0.16	0.044, 0.18
White matter (WM)	461.2 ± 30.8	442.9 ± 33.7	431.5 ± 84.2	0.007, 0.35	0.014, 0.72

## Data Availability

The datasets used and/or analyzed during the current study are available from the corresponding author Maxim Shevtsov on reasonable request.

## Abbreviations

AC	Cyclophosphamide and Adriamycin (chemotherapy regimen)
CAF	Cyclophosphamide, Adriamycin, and Fluorouracil (chemotherapy regimen)
CAP	Cyclophosphamide and Adriamycin (chemotherapy regimen)
CNS	Central Nervous System
DOC	Docetaxel/Paclitaxel (chemotherapy agents)
DWI	Diffusion-Weighted Imaging
FAC	Fluorouracil, Adriamycin, and Cyclophosphamide (chemotherapy regimen)
GM	Gray Matter
ICAM-1	Intercellular Adhesion Molecule 1
MPRAGE	Magnetization Prepared Rapid Acquisition Gradient Echo
MRI	Magnetic Resonance Imaging
PECAM-1	Platelet Endothelial Cell Adhesion Molecule 1
PMES	Post-Mastectomy Syndrome
T1WI	T1 weighted image
TIRM	Turbo Inversion Recovery Magnitude
VolBrain	Automated MRI Brain Volumetric System
WM	White Matter
